# Inclusion of Scar/WAVE3 in a similar complex to Scar/WAVE1 and 2

**DOI:** 10.1186/1471-2121-6-11

**Published:** 2005-03-07

**Authors:** Craig F Stovold, Thomas H Millard, Laura M Machesky

**Affiliations:** 1 School of Biosciences, The University of Birmingham, Edgbaston, Birmingham, B15 2TT, UK

## Abstract

**Background:**

The Scar/WAVE family of proteins mediates signals to actin assembly by direct activation of the Arp2/3 complex. These proteins have been characterised as major regulators of lamellipodia formation downstream of Rac activation and as members of large protein complexes.

**Results:**

We have investigated the interactions of the three human Scar/WAVE isoforms with several previously described binding partners for Scar/WAVE 1 or 2. We find that all three Scar/WAVE isoforms behave similarly and are likely to participate in the same kinds of protein complexes that regulate actin assembly.

**Conclusion:**

Differences between Scar/WAVE proteins are therefore likely to be at the level of tissue distribution or subtle differences in the affinity for specific binding partners.

## Background

Rearrangements of the cortical actin cytoskeleton are essential for numerous cell processes such as cell migration, phagocytosis [1,2], and adhesion [3], in which formation of dendritic networks of polymerised actin plays a key part. The Arp2/3 complex is required in the formation of dendritic networks to provide sites for *de novo *nucleation of actin filaments and to form branch points from existing filaments [4-6]. The mammalian Arp2/3 complex can be activated downstream of Rho family small GTPases by several known interacting proteins, in particular members of the Wiskott Aldrich Syndrome Protein Family (haematopoietic WASP, ubiquitous *N*-WASP, and Scar/WAVE 1, 2, and 3 [Suppressor of Cyclic AMP Receptor mutation/Wiskott Aldrich VErprolin homologous protein]) [7,8]. Homology between these proteins consists of a core proline rich region, and C-terminal WH2 (Wiskott Homology 2) and Acidic (A) domains, of which the WH2 and A domains together are sufficient to activate the Arp2/3 complex [9-11]. *N*-WASP and the Scar/WAVE proteins differ the most at the amino-terminus, designated the Wiskott-Homology 1 (WH1) domain and the Scar/WAVE homology domain (SHD) [9].

Regulation of WASP-family proteins involves many interactions and is still the subject of intensive research. WASP and N-WASP are found in an inhibitory complex with WIP/CR16 proteins and can be activated by the small GTPase Cdc42 and a co-activating protein TOCA-1, recently described by Ho et al. [12]. Phosphorylation on tyrosine residues in the N-terminal half of WASP/N-WASP can also enhance the activation and perhaps serve to prolong its duration ([13-15]). Original reports had suggested that WASP/N-WASP were autoinhibited in their pure form, but whether this form is ever present in cells is unclear, so the physiological relevance of autoinhibition is unclear as well ([16]). Recombinant Scar/WAVE proteins were constitutively active toward Arp2/3 complex *in vitro*, but have been postulated to be regulated by the small GTPase Rac *in vivo *[17,18]. In 2002, Eden et al purified Scar/WAVE1 from bovine brain extracts in association with a complex of four other proteins; p140-SRA1, p125 Nck-Associated Protein (NckAP1), HSPC300, and Abi2 [19]. They proposed a model whereby active Rac could bind to p140-SRA1 and cause it plus NckAP1 and Abi2 to dissociate, releasing active Scar/WAVE in a complex with HSPC300.

Some of the members of this so-called "Scar/WAVE complex" had been previously studied by other groups and implicated in signalling. SRA1 (also called Cytoplasmic FMRP Interacting protein [20]) is a Rac associated protein which also binds to the SH3 domain containing adapter protein Nck, and the Fragile-X Mental Retardation Protein (FMRP) [20-23]. NckAP1 also interacts with active Rac and Nck, although the interaction appears to be indirect in both cases [22,23]. HSPC300 is a small, ubiquitously expressed, uncharacterised Haematopoeitc Stem/Progenitor Cell protein, bearing considerable homology to the Maize actin cytoskeletal associated protein Brk1 [24-26]. Abi2 (Abl interactor 2) is a neuronally expressed SH3 domain containing protein associated with Abl tyrosine kinase [27]. It has a close relative, Abi1, also referred to as e3B1 (eps8 binding protein 1) in early literature [28,29].

Since Eden et al., a similar complex has also been isolated in association with Scar/WAVE2 [30,31]. This complex contains the more ubiquitously expressed Abi1 (as opposed to Abi2) consistent with the more ubiquitous expression of Scar/WAVE 2 compared to the other Scar/WAVE isoforms [31-33]. In these studies, the complex does not dissociate upon Rac activation and is not inhibitory during *in vitro *actin polymerisation assays [31]. Recombinant Abi1 is rather found to increase the Arp2/3 complex activating ability of recombinant Scar/WAVE 2 [31]. Following these studies, the mechanism for regulation of the different Scar/WAVE proteins has been called into question. It has been proposed that differences between Eden et al. and the other two studies may be due to experimental conditions or to genuine differences between Scar/WAVE isoforms [19,30,31,34,35].

Knockdown of Abi1, SRA1 and NckAP1 in mammalian tissue culture cells causes severe defects in formation of lamellipodia [31,36], similar to loss of Scar/WAVE 2 activity [37,38]. Mutations or RNAi knockdown of the only *Drosophila *Scar/WAVE protein severely affects the ability of cultured cells to produce lamellipodia, ruffles and filopodia [39-41]. Mutational and RNAi studies used to produce *Drosophila *cells deficient in the NckAP1 homologue, Kette, reveal a lack of actin based protrusions in the absence of functional protein [42,43]. RNAi knockdown of *Drosophila *Abi1 and SRA1 also prevents the formation lamellipodia in tissue culture cells [39,41,43]. *Dictyostelium *knock out of Scar protein exhibits severe defects in chemotaxis and motility, but cells can still extend pseudopods and migrate directionally [44,45], whereas knockout of the *PirA *gene encoding an homologue of SRA1 causes an excessive lamellipodial protrusion phenotype, thought to be due to unregulated Scar protein activity [46].

While the Scar/WAVE complex is currently thought to be the most likely regulator of Scar/WAVE activity in cells, several other Scar/WAVE binding proteins have been identified and proposed to regulate its activity. IRSp53, for example, binds to Scar/WAVE2 and also to Rac and was proposed to be a Scar/WAVE2 regulator prior to Eden et al. ([47-50]). It was unclear whether IRSp53 binding was specific to Scar/WAVE 2, or ubiquitous among the Scar/WAVEs and where/when Scar/WAVE was associated with IRSp53 as opposed to the other binding partners. IRSp53 is also a scaffold protein, with several partners, raising the possibility of at least two different types of large protein complexes in association with Scar/WAVE proteins [49,51-55].

Scar/WAVE3 is the most tissue-specific of the three mammalian Scar/WAVE isoforms, being found in haematopoietic cells and brain tissue, but not yet being characterised in cells [9,56]. It has not previously been shown whether Scar/WAVE3 interacts with either Abi1/2 or HSPC300. This report presents data indicating that the interaction of Scar/WAVE proteins with Abi1, HSPC300 and IRSp53 is conserved among all three Scar/WAVE proteins, suggesting that multiple different protein complexes likely exist in cells that contain different Scar/WAVE isoforms. This observation suggests that the different Scar/WAVE isoforms are not regulated by exclusive participation in specific complexes, but rather that the regulation is likely to be more subtle, at the level of affinity or modifications/binding partners not yet discovered. Alternatively, Scar/WAVEs may have largely overlapping functions when present in the same cell type, although there is already some evidence against this idea [57].

## Results

### Association of the Scar/WAVE complex with Scar/WAVE 3

**Figure 1:**
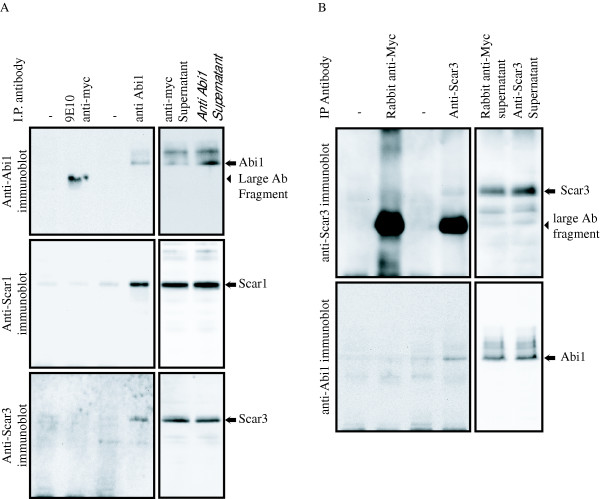
Co-immunoprecipitation of Scar3 and Abi1 from mouse brain extract. Protein G beads were used to precipitate Abi1 from mouse brain extracts in the presence or absence of (A) anti-Abi1 or an unrelated control antibody or (B) anti-Scar3 or an unrelated control antibody. (A) The beads fractions were probed with anti-Abi1, anti-Scar/WAVE1 and anti-Scar/WAVE3. Anti-Abi1 immunoblotting confirmed precipitation of Abi1 only with specific antibody and immunoblotting with anti-Scar/WAVE1 shows association with a known binding partner. Immunoblotting with anti-Scar/WAVE3 revealed Scar/WAVE3 to also be present in Abi1 immunoprecipitates. Anti-Abi1 pulls down both Scar1 and Scar3 together with Abi1. (B) Beads fractions from anti-Scar/WAVE3 immunoprecipitates were probed with both anti-Scar/WAVE3 and anti-Abi1. Both Scar3 and Abi1 are detected in anti-Scar/WAVE3 immunoprecipitates, but not with controls.

Protein sequence conservation between the three Scar/WAVE proteins in the N-terminal Scar Homology Domain (SHD) indicates a potential for association of binding partners with all three members of the family. Scar/WAVE1 and 2 have been shown to associate with the key adapter protein Abi1 through the SHD, so it is possible that Abi1 would also associate with Scar/WAVE3 [19,30]. To test for an interaction between Scar/WAVE3 and Abi1, immunoprecipitations were performed with antibodies recognising Abi1 (Fig. 1A) and antibodies recognising Scar/WAVE3 (Fig. 1B). Anti-Abi1 immunoprecipitates co-precipitate Scar/WAVE1 and 3 with endogenous Abi1 from mouse brain extracts, while Abi1, Scar/WAVE1 and Scar/WAVE3 are not present in immunoprecipitates with an unrelated control antibody or without antibody (Fig. 1A). Abi1 also co-immunoprecipitates with Scar3, but is not detected in the absence of antibody or in unrelated control immunoprecipitates (Fig. 1B). Since Abi1 is thought to bind directly to Scar/WAVE proteins and to connect them with the rest of the Scar/WAVE complex, interaction with Abi1 is a strong indicator Scar/WAVE3 associates in an analogous complex to Scar/WAVE1 and 2 isoforms [35].

### Conservation of Scar complex association between all three human Scar/WAVE isoforms

**Figure 2:**
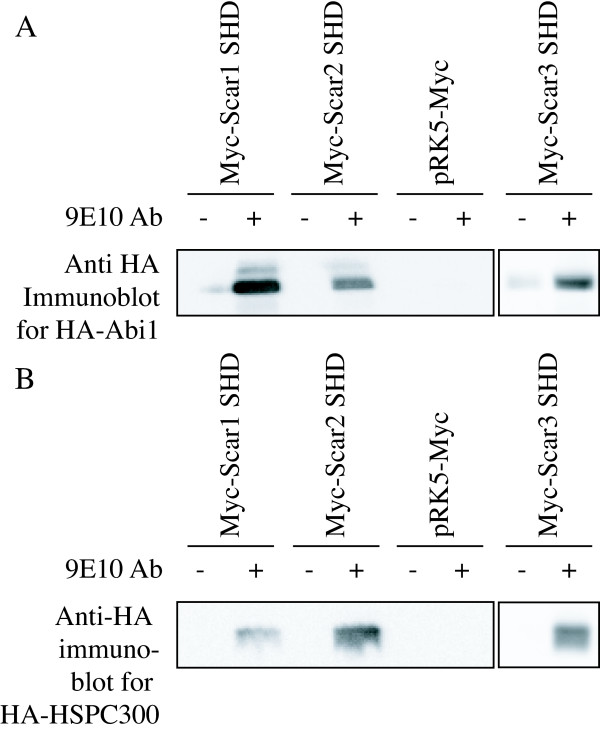
Co-immunoprecipitation of Abi1 and HSPC300 with Scar Homology Domain. Cos 7 fibroblasts transiently transfected as indicated. Protein G beads were used to immunoprecipitate Myc-Scar1 SHD, Myc-Scar2 SHD or Myc-Scar3 SHD from lysates in the presence (+) or absence (-) of anti-Myc (9E10) monoclonal antibody. Empty pRK5-Myc vector was used as an additional negative control. (A) HA-Abi1 was detected in the beads plus antibody fractions for Myc-Scar1 SHD, Myc-Scar2 SHD, and Myc-Scar3 SHD, but not in the negative controls. (B) HA-HSPC300 was detected in the beads plus antibody fraction of immunoprecipitations from cells co-transfected only with Myc-Scar1 SHD, Myc-Scar2 SHD, and Myc-Scar3 SHD, but not empty vector controls or in the absence of 9E10 antibody.

**Figure 3:**
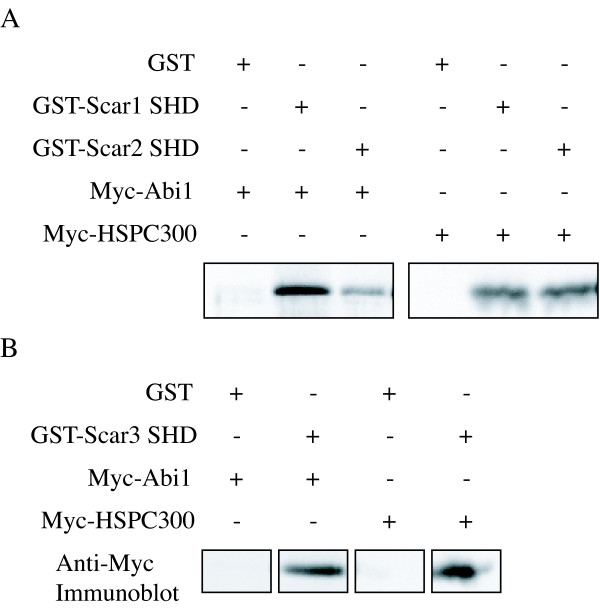
Recombinant SHD pulls down Abi1 and HSPC300. Lysate of Cos7 fibroblasts transfected with HA-Abi1 or HSPC300 was incubated as indicated with GST alone, GST-Scar1 SHD (**A**), GST-Scar2 SHD (**A**), or GST-Scar3 SHD (**B**) on glutathione-s-agarose beads. Beads bound fractions were analysed by SDS-PAGE and immunoblotting with a monoclonal anti-Myc (9E10) antibody. HA-Abi1 and HA-HSPC300 were found in pull-downs with all three Scar Homology Domains, but not with GST alone.

Our data and previously published studies indicate that Abi1 exhibits the potential to bind to all three mammalian Scar/WAVE isoforms. To test whether the association of Scar/WAVE1, 2, and 3 with Abi1 is mediated by the Scar Homology Domain, Myc-Scar/WAVE SHD of each of the three family members were transiently expressed in Cos cells with HA-Abi1 or HA-HSPC300 and immunoprecipitated using a 9E10 anti-myc monoclonal antibody. HA-Abi1 precipitated with each of the three SHDs, but not in the absence of antibody or Myc-SHD (Fig. 2A). HA-HSPC300 also co-immunoprecipitated with all three SHDs, but not the negative controls (Fig 2B). Pull-down experiments performed from HA-Abi1 or HA-HSPC300 transfected Cos cell lysates using GST-fusion proteins of Scar/WAVE1 and 2 (Fig. 3A) and Scar/WAVE3 (Fig. 3B) also show conservation of interaction of all human Scar/WAVE isoforms with Abi1 and HSPC300. Supporting data showing the specific interaction of Abi1 with Scar/WAVE1 SHD is shown in Table 1 and Additional file 1B. Data supporting interaction of Scar/WAVE1 SHD with HSPC300 is shown in Table 1 and in Additional file 1C.

**Table 1:** Yeast two-hybrid analysis of interactions between Abi1, HSPC300, and various domains of Scar1. (+) Indicates a positive interaction backed up by a beta-Gal reporter gene assay. (-) Indicates negative for an interaction between the constructs.

pAct-Scar1	PYTH9	
	
	HSPC300	Abi1
Full Length (FL)	+	+
SB	+	+
SP	+	+
BPWA	-	+/-
PWA	-	-

### Conserved interaction of Scar/WAVE isoforms with IRSp53

**Figure 4:**
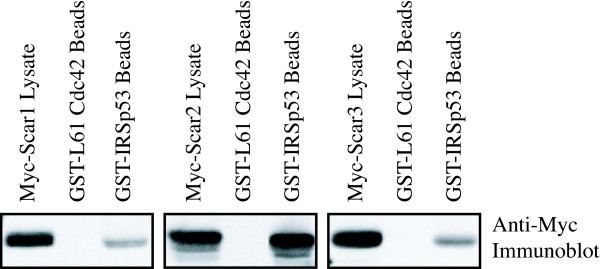
IRSp53 interacts with Scar/WAVE1, 2 and 3. Equivalent amounts of GST-IRSp53 or constitutively active GST-L61 Rac were used for pull-down assays from lysates of Cos cells transfected with Myc-Scar1, 2 or 3. Bead fractions and whole cell lysates, indicating loading with each Scar/WAVE isoform, were analysed by SDS-PAGE and immunoblotting with monoclonal (9E10) anti-Myc antibody. None of the Scar/WAVE isoforms bound to active Cdc42, but all were detected in GST-IRSp53 bead fractions.

Conservation of Scar Homology Domain binding partners raises the question of whether other binding partners of Scar/WAVE are also capable of interacting with the entire Scar/WAVE family. IRSp53 has been implicated in regulation of actin dynamics through binding to Scar/WAVE proteins [49,50,54]. GST-IRSp53 was used for pull down experiments to test for binding to Scar/WAVE1, 2, and 3 (Fig. 4). None of the Scar/WAVE isoforms were detected in the beads fraction of a negative control (constitutively active GST-L61 Cdc42), but all were detected in the beads fraction of GST-IRSp53. An ability to bind to IRSp53 is conserved between all three human members of the Scar/WAVE family, although the binding appears to be strongest for Scar/WAVE2. Conservation of this interaction may indicate that all three Scar/WAVE proteins are regulated by common mechanisms, but the increased binding of Scar/WAVE2 to IRSp53 highlights the possibility that differing affinities for binding partners between the three Scar/WAVE isoforms may affect their roles in cells.

### Cellular localisation of Scar/WAVE3

**Figure 5:**
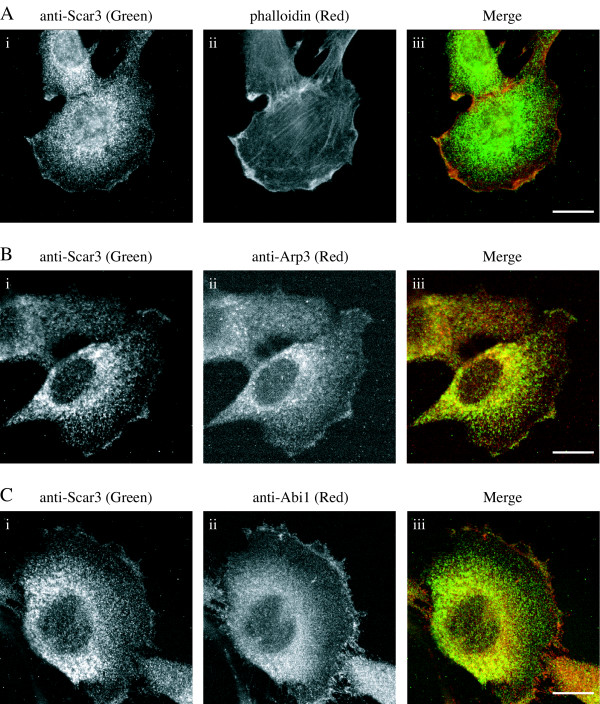
Cellular localisation of Scar3. C2C12 cells were stained with anti-Scar/WAVE3, anti-Arp3, anti-Abi1 and fluorescently labelled phalloidin to investigate the cellular localisation of endogenous proteins. (A) C2C12 cells stained with (i) anti-Scar/WAVE3 (green), (ii) phalloidin (red) (iii) reveal co-localisation of Scar/WAVE3 with cortical polymerised actin in membrane ruffles. Scar/WAVE3 also colocalises with other areas enriched in polymerised actin, but not stress fibres. (B) Co-staining with (i) anti-Scar/WAVE3 and (ii) anti-Arp3 reveals co-localisation of Scar/WAVE3 (green) with the Arp2/3 complex (red) at protruding areas of the cell membrane. (C) Cells stained with (i) anti-Scar/WAVE3 and (ii) anti-Abi1. (iii) Scar/WAVE3 (green) co-localises with Abi1 (red) in some areas of membrane protrusion. A (i), B (i), and C (i) all show a cytoplasmic and perinuclear and cytoplasmic pool of Scar/WAVE3, with enrichment at areas of lamellipodial protrusion. B (ii) and C (ii) show similar patterns of staining for Arp3 and Abi1. Scale bars are equal to 20 μm in all pictures.

As an activator of the Arp2/3 complex, Scar/WAVE3 would be expected to localize to the same areas of the cell as polymerized actin, subunits of the Arp2/3 complex, and members of the Scar/WAVE complex. C2C12 cells were used for immunocytochemistry with anti-Scar3 polyclonal antibody (Fig. 5Ai, Bi, Ci), phalloidin to detect filamentous actin (Fig. 5Aii), anti-Arp2/3 monoclonal antibody (Fig. 2Bii), and anti-Abi1 antibody (Fig. 5Cii). C2C12 cells were used because they were the only standard tissue culture cell line that we found to contain a detectable amount of Scar/WAVE3 protein (see Methods). Scar/WAVE3 mainly exists in a peri-nuclear pool with enrichment to areas of polymerised actin (Fig. 5Aiii). Scar/WAVE3 co-localises with the Arp2/3 complex subunit, Arp3 (Fig. 5Biii), and with Abi1 (Fig. 5iii) in areas of lamellipodial protrusion. Our results show Scar/WAVE3 to localise to areas of polymerised actin near the cell periphery and to co-localise with a known component of the Scar/WAVE complex, indicating that Scar/WAVE3 may have a similar role and method of regulation to Scar/WAVE1 and 2.

### Cellular localisation of Scar/WAVE SHD, Abi1, and HSPC300

**Figure 6:**
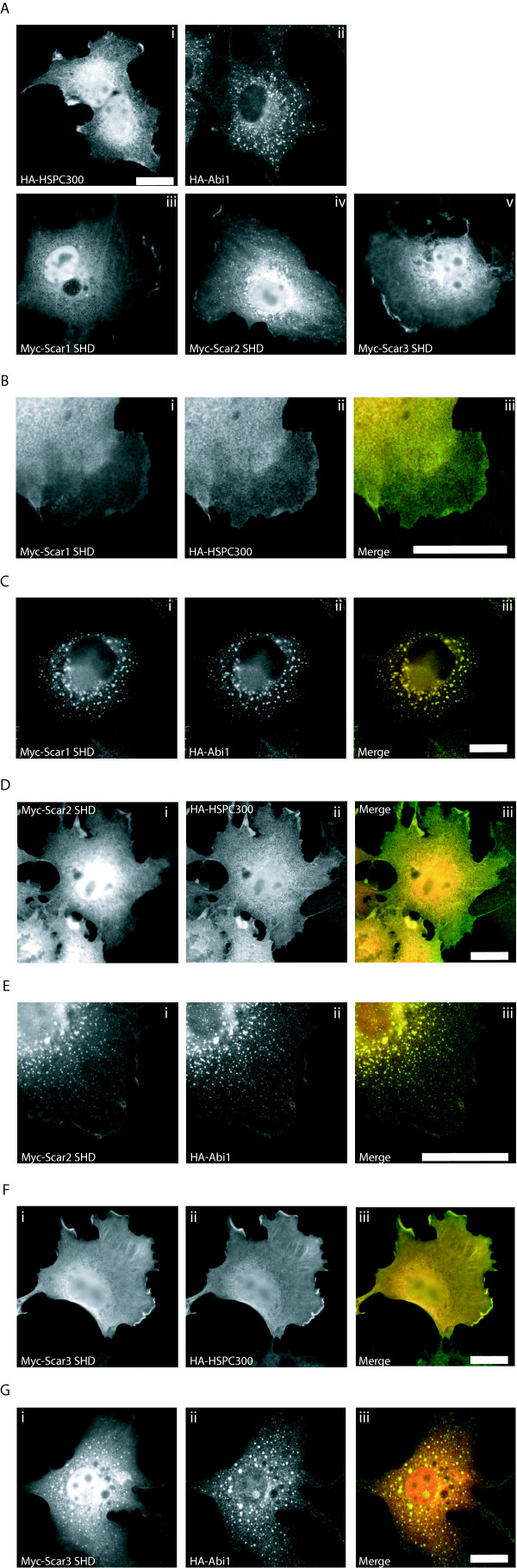
Cellular localization of Abi1, HSPC300, and the Scar homology domain. Transfected Cos cells were stained with a monoclonal anti-Myc antibody and polyclonal anti-HA antibodies to detect over-expressed proteins. (A) Cos7 cells were co-transfected with (i) HA-HSPC300, (ii) HA-Abi1, (iii) Myc-Scar1 SHD, (iv) Myc-Scar2 SHD, or (v) Myc-Scar3 SHD to examine localization of these proteins. HSPC300, and all three SHDs localise in a diffuse cytoplasmic pool with some enrichment at protrusive edges of cells. Abi1 is not detected at the edges of cells, but appears in vesicle-like spots throughout the cytoplasm. (B) Cos7 cells were co-transfected with Myc-Scar1 SHD (i) and HA-HSPC300 (ii) shown as red and green respectively in a merged image (iii). Scar1-SHD and HSPC300 exhibit the same diffuse staining throughout the cytoplasm but also co-localize at protruding edges of cells. (C) Cos7 cells were co-transfected with Myc-Scar1 SHD (i) and HA-Abi1 (ii). (iii) Shows a merge image with myc-Scar1 SHD in red and HA-Abi1 in green. Scar1 SHD and Abi1 both colocalize to punctate spots similar to those seen for Abi1 alone. (D) Cos7 cells co-transfected with Myc-Scar2 SHD (i) and HA-HSPC300 (ii) shown as red and green respectively in a merge image (iii). Both Scar2 SHD and HSPC300 exhibit peri-nuclear staining and localization to protrusive edges of cells. (E) Cos7 cells co-transfected with Myc-Scar2 SHD (i) and HA-Abi1 (ii). (iii) Scar2 SHD (red) and Abi1 (green) show co-localization to cytoplasmic spots and the edges of cells. (F) Cos7 cells co-transfected with (i) Myc-Scar3 SHD (red) and (ii) HA-HSPC300 (green), in a merged image (iii). HSPC300 and Scar3 SHD show diffuse cytoplasmic staining with enrichment at protrusive edges of cells. (G) Cos7 cells co-transfected with (i) Myc-Scar3 SHD and (ii) HA-Abi1. (iii) In a merge image Abi1 (green) and Scar3 SHD (red) co-localize to punctate cytoplasmic spots and to the edges of lamellipodia. Scale bars in all panels are 20 μm.

If the SHD of Scar/WAVE protein mediates the interaction with a regulatory complex it can be expected that the SHD will co-localise with binding partners in cells. Ectopic expression of full length Scar/WAVE isoforms in cells reveals a peri-nuclear and cytoplasmic pool, causing an abundance of polymerised actin in the cytoplasm, but with enrichment of Scar/WAVE protein in lamellipodia [50,58]. A sizeable amount of Myc-Scar 1 SHD (Fig 6Aiii), Myc-Scar 2 SHD (Fig 6Aiv), and Myc-Scar 3 SHD (Fig. 6Av) localises to the cell periphery in lamellipodia, as well as in a cytoplasmic pool similar to full length Scar/WAVE proteins (see Figure 5Ai for comparison). HA-Abi1 is present throughout the cytoplasm in spots (Fig 6Aii), previously described as characteristic of the reticulovesicular system [59]. HA-HSPC300 exhibits a similar cellular localisation to ectopically expressed Scar/WAVE1, 2 and 3 being abundant in and around the nucleus, and diffusely around the cytoplasm with enrichment at lamellipodia (Fig 6Ai).

When expressed with Myc-Scar 1 SHD (Fig. 6Bi), HA-HSPC300 (Fig. 6Bii) co-localises (Shown as yellow in merge pictures [6Biii]) with enrichment in ruffles. Myc-Scar/WAVE 2 (Fig. 6Di), and 3 (Fig 6Fi) SHD also co-localises with HA-HSPC300 (Fig. 6Dii, 6Fii) in the nucleus, cytoplasm, and particularly strongly at areas of ruffles, shown as yellow in merge images (Fig. 6Diii, 6Fiii). All three human isoforms behave the same in the presence of HSPC300 consistent with a conservation of the interaction between Scar/WAVEs and HSPC300.

HA-Abi1 (Fig. 6Cii) strongly co localises with Myc-Scar 1 SHD (yellow, Fig 6Ciii), in unidentified punctate spots (Fig 6Ci). Myc-Scar 2 SHD (Fig. 6Ei) shows a similar co localisation with Abi1 (Fig. 6Eii and 6iii), whilst Myc-Scar 3 SHD (Fig. 6Gi) shows partial co-localisation to Abi1 (Fig. 6Gii and 6iii). Although Myc-Scar3 SHD (Fig. 6Gi) co-localises to the HA-Abi1 spots (Fig. 6Giii) to a lesser extent than Scar/WAVE1 and 2 SHD, leaving some diffuse staining in the cytoplasm and nucleus. Abi1 expression affects the localisation of all three human Scar/WAVE isoforms in Cos7 cells, providing further evidence for a role of the Scar/WAVE complex in regulation of the activity and localisation of Scar/WAVE proteins. The staining pattern seen for overexpressed HA-Abi1 (Fig. 6Aii, Cii, Eii and 6Gii) does not strongly correlate with the cellular localisation of endogenous Abi1 (Fig. 5Cii). Although the punctuate staining pattern seen in cells over-expressing Abi1 has been described as being characteristic of the reticulovesicular system (Ziemnicka-Kotula, 1998), it is also possible that these spots are protein aggregates induced by overexpression of Abi1. The relocalisation of Scar/WAVE1, 2 and 3 SHDs to these vesicles or aggregates may or may not be biologically important, but it does demonstrate the possibility that Abi1 can affect Scar/WAVE protein localisation in cells.

## Discussion

We show that Abi1 and HSPC300 interact with three human Scar/WAVE isoforms, via the Scar Homology Domain. Conservation of Abi1 binding between all three Scar/WAVEs is indicative of the association of the "Scar/WAVE complex" with all three isoforms, as Abi1 seems to be the link between Scar/WAVE and NckAP1 and SRA-1/PIR121 in previous reports [30,31]. Association of the complex with all three Scar/WAVE proteins is a possibility, as all the complex members are widely expressed throughout the body and seem to be found in all tissues where a Scar/WAVE protein is expressed [26,60].

Little is known about the role of Scar/WAVE3 in cells. Expression of the mammalian Scar/WAVE3 gene is largely limited to the brain and lung with relatively little expression in other adult tissues [9,32,56]. The major suggestion has been a role in lamellipodial and filopodial protrusion indicated by localisation in neuronal growth cones and interaction with the Arp2/3 complex [9,57]. Association with a Abi1-NckAP1-SRA1 does indicate a further potential role for Scar/WAVE3 in lamellipodial protrusion given the requirement for these proteins in the ruffling process [31,36]. Scar/WAVE3 is also implicated as a potential tumour suppressor protein in some ganglioneuroblastomas when the gene is down-regulated [56].

The role of interaction of HSPC300 with the Scar/WAVE3 complex is unknown. Loss of function of the *brk1 *gene product, the Maize homologue of HSPC300, causes aberrations in cellular morphology and filamentous actin distribution of leaf epidermal cells [24,25]. This could indicate a role in regulating the localisation of actin polymerisation machinery, but this idea awaits further testing. HSPC300 is also implicated in the frequency of occurrence of renal cell carcinoma [26].

Association of all three Scar/WAVE isoforms with an Abi-NckAP1-SRA1 complex may show that there is some functional redundancy between Scar/WAVE family members but data from mice deficient in a Scar/WAVE isoform indicates that Scar/WAVE1 and 2 do not have completely overlapping functions [37,38,57,61]. The identification of specific binding partners for Scar/WAVE1, such as WRP, BAD or the RII subunit of PKA suggests a potential for differing roles between Scar/WAVE proteins [62-64]. The conservation of the Abi1-NckAP1-SRA1 complex between three Scar/WAVE isoforms suggests that regulation of individual Scar/WAVE proteins is likely to involve components that we have not yet studied [37,57,58,61]. Other proteins that associate with the complex may perform this regulation, or tissue specificity may occur between the complexes formed where differentially expressed related proteins, such as Abi1 or 2, bind to alternative Scar/WAVE isoforms with different affinities. Further study of the Scar/WAVE family and associated proteins will be required to fully understand the true roles and mechanisms of function of these complexes in cells.

## Conclusion

We conclude that Scar/WAVE3 is likely to participate in similar signalling complexes to Scar/WAVE1 and 2 and that the differences between these Scar/WAVE proteins is likely to be at the level of tissue expression, differences in affinity for certain binding partners and possibly interaction with yet undiscovered binding partners.

## Methods

### Reagents and chemicals

All chemicals were purchased from Sigma-Aldrich, UK unless otherwise specified. Antibodies were from the following sources: 9E10 anti-Myc monoclonal (Cancer Research UK), 12CA5 anti-HA monoclonal (Cancer Reaearch UK), anti-Myc polyclonal 1:500 anti-HA polyclonal (Santa Cruz), 1:500 anti-Scar/WAVE3 (Upstate), 1:100 anti-Abi1, or 1:100 anti-Scar/WAVE1 [65], anti-Abi1 (gift from Giorgio Scita, Instituto Europeo di Oncologia, Milan, Italy), HRP-conjugated secondary antibody anti-mouse or anti-rabbit (Jackson labs), anti-Arp3 (Sigma Israel), Goat anti-mouse or rabbit, FITC, TRITC, Alexa-488 or Alexa-546 Conjugates (Molecular Probes). The specificity of the anti-Scar/WAVE3 antibody for Scar/WAVE3 has been demonstrated by Oda and colleagues [47].

### Vectors and cloning

HSPC300 and Abi1 expression vectors were created using the Gateway Cloning system (Invitrogen). Open reading frames for HSPC300, and Abi1, derived from I.M.A.G.E. clones 4519512 (HSPC300) and 4158413 (Abi1) (UK-Human Genome Resource Centre, Babraham), were amplified by PCR and ligated into the pENTR-Topo entry vector (Invitrogen). N-terminally HA- and Myc- tagged HSPC300, and Abi1, were created in pRK5 DEST-Myc or pRK5 DEST-HA Gateway destination vectors by recombination from entry vectors. pRK5 DEST-Myc or pRK5 DEST-HA were created by ligation of the Gateway Vector Conversion System (Invitrogen) reading frame A cassette into pRK5-myc or pRK5-HA cut with SmaI. Scar/WAVE1, Scar/WAVE2, and Scar/WAVE3 full length and deletion constructs were created by PCR and cloned into pRK5-myc or pGEX4T-2. Scar/WAVE-1 constructs were derived from KIAA00429[7]; Scar/WAVE2 constructs were derived from pDSRed Scar2, a kind gift from Giorgio Scita (Innocenti et al., 2004); Scar/WAVE3 constructs were derived from pcDNA Scar3, a kind gift from John Scott, Portland, U.S.A. L61 Cdc42-pGex2T and IRSp53-pRK5-Myc were a gift from Alan Hall (LMCB, London) [54]. IRSp53 was subcloned into pGex4T-2 using BamH1 and EcoR1 restriction sites. For Yeast Two-Hybrid analyses, Abi1 and HSPC300 were fused in-frame to the C-terminus of the GAL4 DNA-binding domain in pYTH9. Myc-tagged Scar/WAVE1 deletion constructs have been previously described[7]. Scar1 deletion mutants (described in Additional file 1) were fused to the C-terminus of the GAL4 activation domain in pACT-II [66]. Yeast 2-hybrid analyses were performed as previously described [66].

### Cell culture, transfection, and lysis

Cos 7 and C2C12 cells were grown in DMEM plus 10% Foetal Calf Serum and antibiotics. Transfections were performed using Genejuice transfection reagent (Novagen) following the manufacturers instructions. Cell lysis was performed from confluent cells in a 1% Triton X-100 lysis buffer (1% Triton X-100, 50 mM Tris-HCl pH 7.5, 150 mM NaCl,, 1 mM EDTA, 10% Glycerol, 1 mM PMSF, and 1 μg/ml each of chymostatin, aprotinin, leupeptin, and pepstatin). Lysates were cleared by centrifugation and equalised to 1 mg/ml total protein concentration.

### Preparation of mouse brain extract

Mouse brains were homogenised on ice in 2 μl of 1% Triton X-100 lysis buffer per mg of tissue using a fine gauge needle. Homogenates were centrifuged once for 10 minutes at 13,000 rpm in a microcentrifuge, then for 30 minutes at 100,000 g in a Beckmann TLA-100.2 rotor. Supernatants were equalised to 1 mg/ml total protein concentration with 1% Triton X-100 lysis buffer.

### Immunoprecipitations

Lysates or brain extracts (500 μg of total protein) were incubated with 30 μl bed volume of pre-washed protein G beads (Cancer Research UK) for 1 hour, designated beads minus antibody controls. Beads were precipitated and supernatants removed. 5 μg of antibody monoclonal 9E10 anti-myc, 12CA5 anti-HA, Rabbit anti-Scar/WAVE3, or Mouse anti-Abi1 or an irrelevant control antibody (rabbit or mouse anti-myc) was added to the supernatant and incubated for 1 hour, before the addition of 30 μl bed volume of pre-washed protein G beads, designated beads plus antibody, for 1 hour. Beads were precipitated, and the supernatant removed. Beads were washed 3 times in 30-fold bed volume of lysis buffer. Beads were resuspended in an equal volume of 2× SDS-PAGE loading buffer [67]. 10 μl of beads sample and supernatant was analysed by SDS-PAGE and western blotting.

### GST-production and pull downs

GST-fusion proteins were expressed in BL21 *E.coli *induced for 3 hours with 0.2 mM IPTG, or 15 hours at 25°C for GST-IRSp53. Cells were spun down and sonicated in 1 ml of 1% Triton PBS (1% Triton X-100, PBS pH 7.5, 1 mM PMSF, and 1 μg/ml each of chymostatin, leupeptin, aprotinin, and pepstatin) per 100 ml bacterial culture. GST fusion proteins were batch purified on 100 μl Glutathione-s-agarose (Sigma) per 100 ml culture and washed 5 times with 10-fold bed volume of sonication buffer. Beads were resuspended in an equal volume of 1% Triton lysis buffer. SDS-PAGE analysis and coomasie staining were used to qualify GST fusion proteins. 500 μg total protein of transfected Cos7 cell lysate was incubated with GST-fusion protein or GST alone for 1 hour. Beads were precipitated, lysates removed and the beads were washed 3 times in 1% Triton lysis buffer. Beads were resuspended in and equal volume of 2× SDS-PAGE loading buffer. Samples were analysed by SDS-PAGE and western blotting.

### SDS-PAGE and Western blotting

Proteins were separated by SDS-PAGE and transferred onto nitrocellulose membrane by western blotting. Blots were saturated with 5% dried milk in PBS 0.2% Tween-20, probed with 1:500 9E10 anti-Myc monoclonal, 1:500 12CA5 anti-HA monoclonal, 1:500 anti-Myc polyclonal 1:500 anti-HA polyclonal, 1:500 anti-Scar/WAVE3, 1:100 anti-Abi1, or 1:100 anti-Scar/WAVE1 primary antibody in 2.5% BSA 0.2% Tween-20 PBS followed by 1:10'000 dilution of HRP-conjugated secondary antibody diluted in 0.2% Tween-20 PBS. Bound secondary antibody was visualised using SuperSignal (Pierce) according to the manufacturers instructions.

### Immunofluorescence microscopy

Cells cultured on glass coverslips were fixed in 4% paraformaldehyde and permeabilized with 0.1% Triton PBS. Cells were stained with 1:200 Rb anti-HA polyclonal, 1:200 12CA5 anti-HA monoclonal, 1:200 9E10 anti-myc monoclonal, 1:100 anti-Scar\WAVE3, 1:200 monoclonal anti-Arp3, or 1:25 anti-Abi1 primary antibodies diluted in 1% BSA PBS. Secondary antibodies used were Goat anti-mouse or rabbit, FITC, TRITC, Alexa-488 or Alexa-546 Conjugates at 1:200 dilution in 1% BSA PBS. Filamentous actin was visualised using Alexa-546 conjugated phalloidin (Molecular Probes) diluted 1:500 in 1% BSA PBS. Slides were viewed using a BioRad MRC100 confocal laser scanning microscope. For endogenous Scar/WAVE3 labelling, it was important to test which cell lines expressed Scar/WAVE3. Among the following cell lines: NIH 3T3, N1E 115 neuroblastoma, N19 neuroblastoma, PC6 neuronal precursor, J774.A1 bone marrow macrophage, RAW 264.7 alveolar macrophage and C2C12 myoblast, we found that only C2C12 expressed endogenous Scar/WAVE3 by western blotting of whole cell lysates (M. Vartiainen, unpublished results). We thus used C2C12 for localisation of endogenous Scar/WAVE3 protein.

## Abbreviations

GST glutathione-S-transferase, WH2 WASP-homology 2, A acidic sequence motif, SHD Scar homology domain, NckAP1 Nck-associated protein 1, HSPC300 haematopoeitc stem/progenitor cell protein, Abi 2 abelson tyrosine kinase interactor 2

## Authors' contributions

C.F.S. performed most of the experiments and contributed intellectually to the design and interpretations. C.F.S. also drafted the manuscript. T.H.M. performed the experiments shown in Figure 4 and contributed intellectually to the project as a whole. L.M.M. Contributed to the design and analysis of the experiments and data and edited the manuscript.

## Supplementary Material

Additional File 1 Characterization of HSPC300 and Abi1 binding domain of Scar/WAVE1.  (**A**) Schematic representation of the domain architecture of Scar/WAVE family proteins and the deletion constructs used in these experiments.  Numbers indicate start or end point of Scar/WAVE1 deletion mutants in amino acid residue position.  Start/End points for Scar/WAVE2 deletion mutants are FL = 498, delta A = 456, SPW = 455, SP = 435, SB = 245, SHD = 170, BPWA = 171, PWA = 246.  Start/End points for Scar/WAVE3 deletion mutants are FL = 499, delta A = 488, SPW = 457, SP = 439, SB = 238, SHD = 171, BPWA = 172, PWA = 239.  FL; Full length.  SHD; Scar Homology Domain.  B; Basic Rich Region.  PRR; Proline rich region.  W; Wiskott Homology 2 domain.  C; Central or connecting region.  A; Acidic Rich Region.  (**B**) Co-immunoprecipitation of HA-Abi1 with Myc-Scar/WAVE deletion constructs.  (**C**) Co-immunoprecipitation of HA-Abi1 or HA-HSPC300 respectively with Myc-Scar/WAVE deletion constructs.  Protein G beads were used to immunoprecipitate protein from transiently transfected Cos 7 fibroblasts before (-Ab) and after addition (+Ab) of an anti-Myc (9E10) monoclonal antibody.  Bead bound fractions and supernatants (Sup) were analysed by SDS-PAGE followed by immunoblotting.  Blots were probed with an anti-HA polyclonal antibody for the presence of HA-Abi1 or HA-HSPC300 in immunoprecipitated complexes. Click here for file
